# Human babesiosis, an emerging tick-borne disease in the People’s Republic of China

**DOI:** 10.1186/s13071-014-0509-3

**Published:** 2014-11-18

**Authors:** Xia Zhou, Shang Xia, Ji-Lei Huang, Ernest Tambo, Hong-Xiang Zhuge, Xiao-Nong Zhou

**Affiliations:** National Institute of Parasitic Diseases, Chinese Center for Disease Control and Prevention, WHO Collaborating Centre for Malaria, Schistosomiasis and Filariasis; Key Laboratory of Parasite & Vector Biology, Ministry of Health, Shanghai, 200025 People’s Republic of China; Department of Parasitology, Medical College of Soochow University, No.199 Renai Road, Suzhou, 215123 People’s Republic of China; Biochemistry Department, Centers for Sustainable Malaria Control, Faculty of Natural & Agricultural Sciences, University of Pretoria, Pretoria, South Africa

**Keywords:** Human babesiosis, *Babesia*, Emerging disease, P.R. China

## Abstract

Babesiosis is a typical zoonotic, emerging disease caused by a tick-borne intraerythrocytic protozoan of *Babesia* spp. that also can be transmitted by blood transfusion. Babesiosis imposes an increasing public-health threat. We reviewed and mapped epidemiological studies on *Babesia* in vectors and/or rodents in the People’s Republic of China (P.R. China) and found that *B. microti* was the predominant species detected in the investigated regions such as Heilongjiang, Zhejiang, Fujian provinces and Taiwan island. We reviewed a series of sporadic human babesiosis cases collected from 1940’s to 2013, in Yunnan, Inner Mongolia, Taiwan and Zhejiang and other regions including a main endemic area of malaria on the China-Myanmar border areas in P.R. China. Clinical manifestations of human babesiosis were also reviewed. Human babesiosis may have previously been overlooked in P.R. China due to a lack of medical awareness and the limitation of clinical diagnostic methods.

**Electronic supplementary material:**

The online version of this article (doi:10.1186/s13071-014-0509-3) contains supplementary material, which is available to authorized users.

## Review

Babesiosis is an intraerythrocytic parasitic and zoonotic disease, caused by *Babesia* spp*.* in humans and animals worldwide. *Babesia microti*–like organisms have been reported to cause illness in Japan, People's Republic of China (P.R. China) and other Asia-Pacific regions. In USA, most documented cases are caused by *B. microti*, and in Europe by *B. divergens* [1]. Babesiosis is also gaining increasing attention as a potential emerging tick-borne zoonosis which can also be transmitted by blood transfusion [2-5]. P.R. China is located in both northern and eastern hemispheres and the distinctive characteristic of China’s climate is its variety. The northern region has a subarctic climate, whereas the southern area is dominated by tropical weather, and the climate variability would undoubtedly affect the vector-borne diseases [6,7]. During the last decade new tick-borne infections have emerged and the incidence rate has been rising steadily in several regions of China [8-15]. The latest research indicated that there is co-prevalence of *B. microti* infections and malaria on the China-Myanmar border areas in Yunnan province in south of P.R. China [16]. Recent studies of cytokine activation and erythrocyte cytoadherence in babesiosis and malaria have exploited these similarities to provide new insights into malaria pathobiology [17]. Meanwhile, the latest information on human babesiosis in P.R. China was examined and the primary question we wanted to address was whether this zoonosis has been sporadic or more widespread than previously appreciated. Our review highlights the challenges of medical awareness of the disease, diagnostic tools and techniques in microbial detection methods, and recommends innovative strategic measures for prevention and control of human babesiosis.

### Clinical features, pathogenensis, and diagnosis of babesiosis and malaria

**Figure 1:**
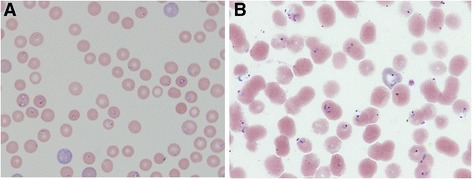
**Giemsa-stained thin blood smears showing the similarities of the intraerythrocytic ring form stages of** ***B. microti*** **and** ***P. falciparum*** **, showing an intraerythrocytic ring form stage of** ***B. microti*** **(thick arrow) and ring form stage of** ***P. falciparum*** **(thin arrow); Original magnification × 1,000.** (**A**. Ring form stage of *B. microti*, **B**. Ring form stage of *P. falciparum*).

Both *Plasmodium* and *Babesia* species are intraerythrocytic protozoans and elicit similar inflammatory responses [17] with similarities in clinical manifestations including headaches, fever, chills, nausea, vomiting, myalgia, altered mental status, disseminated intravascular coagulation, anaemia with hypotension, respiratory distress, hepatomegaly and renal insufficiency. All symptoms are common to both diseases [18]. Traditionally, malaria cases can be diagnosed from a consideration on the basis of a travel history and a careful microscopic or molecular assay [19]*.* However, the ring forms of *Plasmodium falciparum* may be difficult to distinguish from the *Babesia* spp. (Figure 1). Although tetrads of merozoites that are arranged in a cross-like pattern are pathognomonic and provide a typical morphology for diagnosis of babesiosis caused by *B. microti,* it rarely appears as tetrads in human erythrocytes [20].

An infant from the Ivory Coast was suspected of being infected by *Babesia sp.* in Africa who at first was treated as having malaria [21]. Similar co-infection with both agents were reported from experiments using animal models of malaria, the rhesus monkey imported from Guangxi, P.R. China with a suspected infection of *B. microti*-like that originated from a primate-breeding facility [22]. Two cases of febrile patients who were co-infected with *Plasmodium* and *Babesia* in malaria endemic areas were detected in our laboratory using molecular epidemiology survey, but the dominant parasite was unclear and to detect species of *Plasmodium* or *Babesia* by microscopy in co-infection cases was more difficult [16]. The pathobiology of malaria has been extensively studied in humans; related research on babesiosis is still insufficient. Investigation of similarities and differences in the pathogenesis of babesiosis and malaria could lead to additional fundamental insights for both conditions. Continued investigation of similarities and differences in the pathogenesis of babesiosis and malaria could have broad implications relating to evolutionarily conserved mechanisms of host cell entry in these related apicomplexan parasites and may pave the way toward a detailed molecular understanding of erythrocyte invasion of both pathogens.

In 2005, Lobo *et al.* reported that *B. divergens* and *P. falciparum* use the common receptors, glycophorins A and B, to invade the human erythrocyte [23]. Duivenvoorde LM *et al.* reported that a rhesus macaque chronically infected with *B. microti* was able to control the infection with *P. cynomolgi* better than naïve monkeys [24]. The suppression of a *Plasmodium* infection after chronic exposure to *B. microti* warrants further investigation for a possible protective role of *Babesia* infection on *Plasmodium*.

Babesiosis, recognized as an important disease of domestic animals and more recently as an emerging health problem in humans, is caused by related intraerythrocytic protozoa with a similar pathogenesis and a clinical path.

### Current challenges in epidemiological investigation and clinical diagnosis

Due to the low level parasitemia of babesiosis in the early course of illness, it has been suggested that at least 300 microscopical fields should be reviewed before considering a blood smear free of *Babesia*. This puts limitations of microscopy diagnosis of babesiosis to be used in the field. Broadly specific PCR for *Babesia* allows identification of *Babesia* spp. and differentiate these from *Plasmodium* infections, hence these tools should be applied to confirm the identity of the pathogens [16,25], however, the molecular methodology is relatively difficult to use in the field surveys because it is more expensive and requires a skilled technologist [26].

All of these three confirmed *B. microti*-infection patients, either by investigations of smears and/or *Babesia* specific PCR [25,27] proved that they are very useful tools not only to confirm the correct diagnosis of infection of *B. microti*, but also to monitor the patient’s response to antiparasitic therapy.

The most probable mode of infection was from tick bites due to the patients’ extensive outdoor recreational activities. One patient received blood products just prior to infection which suggested that the *B. microti* might have been transmitted by blood transfusion [28]. If this was the case, screening of blood donors in this region is urgently needed.

Major problems associated with diagnosing *Babesia* spp. infections in humans include the general lack of clinical awareness of babesiosis in the medical community, the non-specific clinical manifestations, and the absence of simple and effective rapid diagnostic test (RDT). Convenient, well-evaluated diagnostic tools such as serological tests or molecular biological assays designed for rapid and reliable detection of such pathogens are not yet readily available to most routine diagnostic laboratories [4]. Additionally, conventional laboratory test-results in clinical cases of human babesiosis may be non-specific, such as high levels of transaminases, alkaline phosphatases, unconjugated bilirubin, and lactic dehydrogenase in addition to normochromia, normocytic anaemia, and thrombocytopenia. Occasionally, leucopenia may also be present, probably owing to a tumour necrosis factor (TNF)-mediated immune response similar to that seen in severe cases of malaria [29].

On the immunological diagnosis of babesiosis, indirect immunofluorescence assay (IFA) is regarded as the standard assay for the detection of *Babesia* antibody [30]. Another immunological assay by immunoblot for detection of *B. microti* antibody is also available [31]. Luo Y. *et al.* [32] identified a novel secreted antigen designated as *B. microti* secreted antigen 1 (BmSA1) and found BmSA1 could be a promising and universal target for the serodiagnosis of human babesiosis and for an epidemiological survey. Ooka H. *et al.* [33] applied an ELISA using rBmP94/CT for diagnosis of *B. microti* infection, and it demonstrated high sensitivity and specificity when tested with the sera from mice experimentally infected with *B. microti* and other species of *Babesia*. These results indicated that BmSA1 and BmP94/CT could be potential markers for surveillance of human babesiosis caused by *B. microti*. However, to date, serological studies on population tick exposure to *Babesia* have not been conducted and the diagnostic value of these antigens to human babesiosis has not been evaluated. In New England (Country) where the disease is enzootic, to evaluate acute babesiosis cases, an immunofluorescent assay (IFA) was carried out. The sensitivity of the IFA was 91%, the specificity was 99%, the positive predictive value was 86%, and the negative was 99%. So IFA should be a sensitive, specific routine clinical diagnosis of acute and convalescent babesiosis [34]. The latest survey on seroprevalence of blood donors was carried out in *Babesia* endemic areas of the Northeast and Upper Midwest in the USA. The presence of antibodies against *B. microti* was tested by using an IFA. The results showed that 2% (42/2150) of the donors were positive and one patient was confirmed to have an ongoing infection of *Babesia* by positive PCR (1/42) [35]. The ELISA survey on the febrile cases were applied by diagnostic antigen of BmSA1 expression and purified in our group to conduct the prevalence of the human babesiosis. The results showed that BmSA1 have cross-reactivity with malaria cases in China. The former seroprevalence survey in *Babesia* endemic areas of the Northeast and Upper Midwest in the USA showed that most of the sero-positive cases (41/42) were negative in the molecular survey. Thus, clinical diagnosis of human babesiosis can be further complicated by persistent low parasitemia or asymptomatic latent infections, particularly in malaria and babesiosis syndemic areas.

### Evidence for occurrence of vectors, reservoir hosts and pathogenic *B. microti* in P.R. China

There are many babesiosis natural foci areas where *Babesia* spp. in ticks or/and other reservoir hosts are found in P.R. China. *B. microti*-like rodent parasite was isolated from the tick, *Ixodes persulcatus,* and collected from the northern forest area of Heilongjiang province, P.R. China [36]. Field rodent surveys for *Babesia* infections performed from 2002 to 2005 in the vicinities of human babesiosis cases in southeastern of P.R. China confirmed the presence of Kobe strain of *B. microti* in rodents from Zhejiang, Fujian and Taiwan [37]. In 2012, Jiang *et al.* [38] carried out a survey on the infection of *Babesia* protozoa in rodents in Chun’an County, Zhejiang province and reported cases of human babesiosis using molecular detection. The molecular survey demonstrated that *B. microti* infections were present in two *Rattus tanezumi* and one *R. norvegicus*. The results were in accordance with the distribution of *B. microti* in the vicinities where human babesiosis occurred in the P.R. China [14].

**Figure 2:**
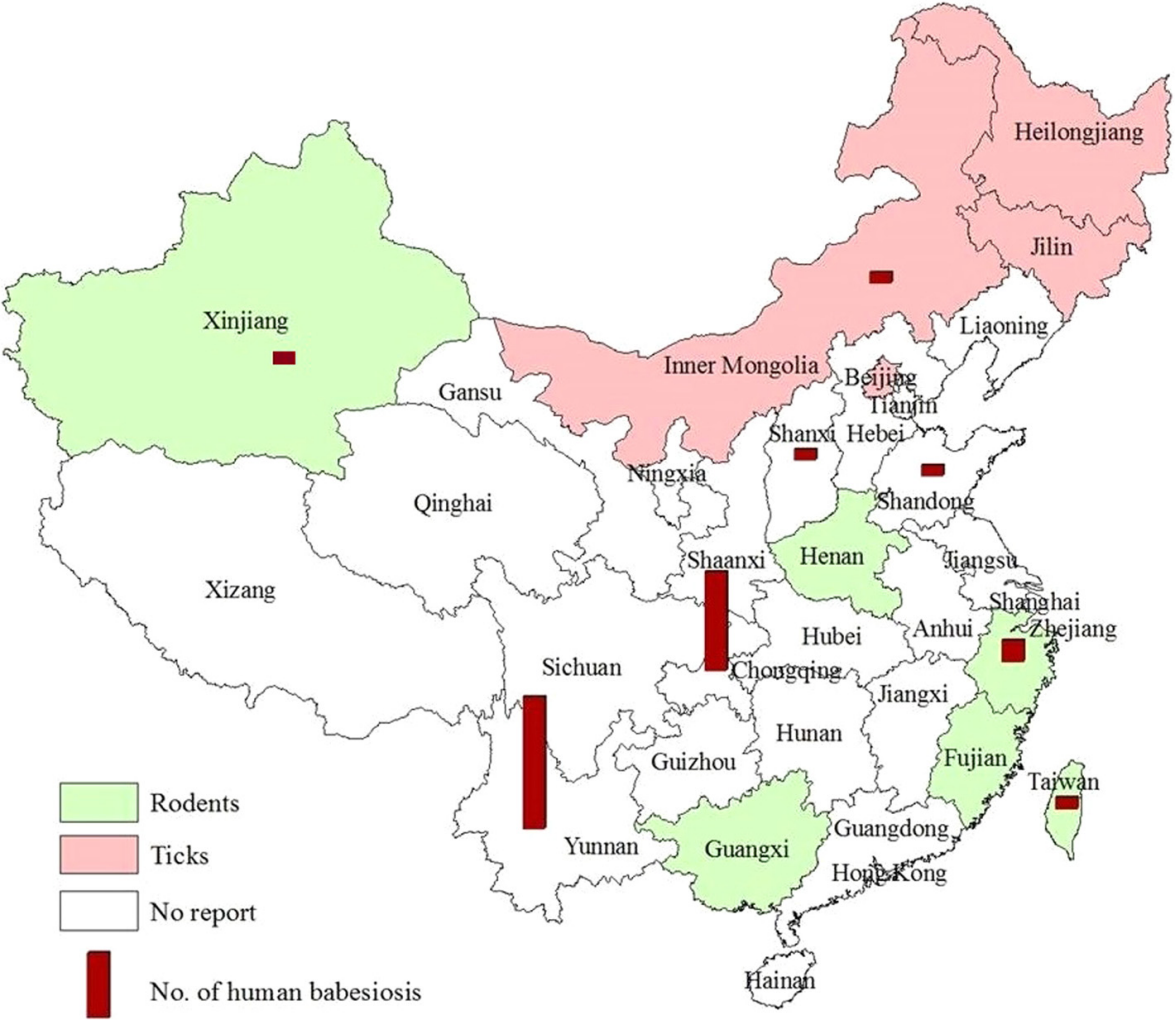
**Geographic areas where endemic areas of human babesiosis and ixodes tick vectors or wild rodents infected with** ***B. microti*** **in China.** Light green colors denote areas where the rodents that acted as the reservoir hosts in transmitting *Babesia* were detected; Pink colors denote areas where ixodes tick vectors transmitting *Babesia* were detected; Red pillars denote areas where human babesiosis reported, height of the pillars denote the number of reported babesiosis cases. White colors designate areas where no survey been carried on *Babesia* in ticks or rodents, despite some areas with reported human babesiosis cases.

*I. persulcatus* is regarded as the most important vector for human tick-borne diseases in P.R. China and there is anecdotal evidence that *I. persulcatus* can transmit *B. microti* and *B. divergens* to both humans and animals [1]. A molecular survey in Heilongjiang province showed that of all the obtained *Babesia* infections in *I. persulcatus*, 78% of ticks were infected with *B. microti*; the remaining 22% with *B. divergens.* The second important tick, *Haemaphysalis concinna*, 82% were infected with *B. microti* and 18% of *B. divergens* [39,40]. Another molecular survey carried out in Jilin province revealed that *B. microti*-infections were the main strain in *I. persulcatus* with an infection rate of 5% (20/379) [41], indicating that *B. microti* may be the dominant *Babesia* species in northeastern regions of China. Furthermore, the molecular survey in Inner Mongolia Autonomous Region demonstrated that *Dermacentor nuttallia* was the predominant tick species with a 66% (29/44) rate of infection with *B. divergens* and 34% (15/44) with *B. microti* [42]. A molecular survey by the broadly specific primers for hemoprotozoa in *Piroplasma* on the tick-borne pathogen infection rate in wild rodents in eight sites in Xinjiang Uygur Autonomous Region was carried out. Of all the positive infections of *Babesia* or *Theileria* (53/165) samples, only 6% (3/53) of the wild rodents were confirmed to be infected with *B. microti,* and the remaining were infections of *Theileria* spp. This indicates that *B. microti-*infections were not the predominant species in Xinjiang Uygur Autonomous Region [43]. In 2008, *B. microti*-like parasites were identified in one rhesus monkey (*Macaca mulatta*) imported from Guangxi Zhuang Autonomous Region and the suspected infection of *B. microti* originated from a primate-breeding facility [22]. This provided anecdoctal evidence of the presence of natural foci of *B. mciroti* in Guangxi Zhuang Autonomous Region. *B. microti* was also detected from *I. persulcatus* in Beijing and the partial sequences of 18S RNA gene of *B. microti* was submitted to NCBI GenBank (accession no. JX962781). Recently, a total of 33 rodents (22 *Apodemus agrarius* and 11 *R. norvegicus*) were captured from Xinyang City, Henan province. Inoculation experiments were carried out in mice and indicated that one of the rodents was co-infected with *A. phagocytophilum* and *B. microti* [44]. Thus, the detection and isolation of both pathogens from the single rodent in Henan province further highlighted the possibility of co-infection in human beings and should alert public health. In summary, previous epidemiological studies on *Babesia* infections in vectors or rodents by universal primers for *Babesia* spp. has shown that the occurrence of pathogenic *Babesia* spp. exists widely in P.R. China. Specific PCR confirmed that *B. microti* infections were the predominant species in southeastern and northeastern of P.R. China while *B. divergens* and other *Babesia* species may be the main pathogen genus of *Babesia* in Inner Mongolia [42] and Xinjiang Uygur Autonomous Region of P.R. China [43] (Table 1 and Figure 2).

**Table 1:** **Positive ratio of** ***B. microti*** **detection in reservoir hosts or ticks in China**

		**Species of hosts or vectors**		**Infection rate, % (Positive no./examined no.)**	**Reported human babesiosis cases**	**References**
**Province**	**Site**	**Ticks**	**Rodents**			
Heilongjiang	Mohe port Suifenhe port	*Ixodes persulcatus, Haemaphysalis concinna*	N/A^※^	3.4 (13/383)	No	Yang LW *et al*. [40]; Fu WM *et al*. [39]
			N/A			
Jilin	Changbai port	*I. persulcatus*	N/A	5.3 (20/379)	No	Pu Y *et al*. [41]
Beijing	/	*I. persulcatus*	N/A	N/A	No	NCBI, 2011 GenBank, no.JX962781
Inner Mongolia	Ceke port,Mandula port, Manzhouli port	*Dermacentor nuttallia*	N/A	1.2 (15/1303)	Yes	Hao GF *et al*. [42]
Guangxi	/	N/A	*Macaca mulatta*	N/A	No	Voorberg-vd Wel. A *et al*. [22]
Henan	Xinyang	N/A	*Rattus norvegicus*	9.1 (1/11)	No	Zhao X *et al*. [44]
Zhejiang	Hangzhou	N/A	*R. tanezumi, R.norvegcus*	2.8 (3/106)	Yes	Jiang LP *et al*. [38]
	Tiantai Mountain	N/A	*Niviventer confucianus*	50.0 (4/8)	Yes	Saito-Ito *et al*. [37]
Fujian	Wuyi Mountain	N/A	*N. confucianus*	33.3 (6/18)	No	Saito-Ito *et al*. [37]
Taiwan	Nantou, Gaohsiung	N/A	*R. coxinga, Citellus horsefieldii*	5.1 (2/39)	Yes	Saito-Ito *et al*. [37]
Xinjiang	Wuchai wan, Fuhai, Bulzin, Hanashi Lake	N/A	*C. erythrogenys Lagurus luteus*	1.8 (3/165)	Yes	Zamoto *et al*. [43]

^※^N/A, not applicable.

### Evidence of *B. microti* infection in humans and non-human primates in China

Human babesiosis is characterized as an emerging disease and the severity of *Babesia* infection is variable, ranging from an asymptomatic infection to severe life threatening disease depending on the host immune status and species of infecting *Babesia*. Severe babesiosis generally occurs in patients who are immune-compromised including the persons who are over 50 years old, with malignancy, HIV or immunosuppressive medication [1]. Current limitations of light microscopy diagnosis make it difficult to differentiate the ring forms of *P. falciparum* and *Babesia* spp. clinically [1]. The co-prevalence of *B. microti* infections and malaria on the China-Myanmar border areas in Yunnan province of southwestern China during April, 2012 to June, 2013 were found in farmers who involved in outdoor activities in the forests or mountains, or in individuals with other underlying diseases and/or have had blood transfusion or other blood products [16]. The investigation carried out in Europe demonstrated that less than 50% of tick bites are documented in individuals who commonly do professional or recreational outdoor activities, and frequently suffer from several tick-bites each year [45]. As such, the prevalence of pathogens in ticks and tick exposure are important risk factors for acquiring infections with tick-borne organisms, which may explain the significantly higher seroprevalence in high risk groups compared with blood donors [46,47]. The prevalence of *Babesia* in ticks and rodents and sporadic human babesiosis cases, have been investigated or reported in several provinces or regions in P.R. China (Figure 2).

A series of surveys in areas including Xinjiang Uygur Autonomous Region [43], Heilongjiang province [36,39,40], Jilin province [41], Inner Mongolia Autonomous Region [42], Henan province, Zhejiang province [38], Fujian province, Taiwan [37], and Guangxi Zhuang Autonomous Region [22] demonstrated the presence of *Babesia* in ticks or animals (Figure 2). These areas investigated above appear to be natural foci of piroplasms and may present a hazard to public health. Hence, systematic studies on seroprevalence in the population should be carried out in potentially infected humans in P.R. China.

**Figure 3:**
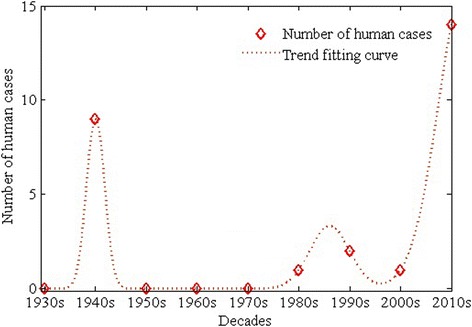
**Human babesiosis cases reported in China from 1930s up to 2013.** Tread fitting curve denotes the development of human babesiosis cases from 1930s to 2014. Totally, assuming the cases reported by Hung *et al.* were human babesiosis, about 27 babesiosis cases that have been reported in China. About a third of the 27 babesiosis cases that have been reported in China were occurred before 1980 which were focused during 1940s and two thirds thereafter.

During 1931 to 1944, Hung S.L. *et al.* [48] produced a series of reports on human parasitemia in Beibei, Chongqing, P.R. China, with the protozoa present in human erythrocytes described as being similar to ring form stages of *P. falciparum* but having fundamental differences in size (smaller) and no pigment, therefore strongly indicating that they were *Babesia*. These reports were available 13 years earlier than the first case describing human babesiosis reported in a Yugoslavian farmer in 1957 [49], and may be the first report of human babesiosis. In 1969, the first case of human babesiosis caused by *B. microti* spp. with *I. scapularis* as vectors was reported in an immunocompromised patient on Nantucket Island, off the coast of Massachusetts in USA [50]. Sporadic human babesiosis cases have been documented in Yunnan, Inner Mongolia, Taiwan, Zhejiang, Shanxi and other regions in China from 1984 to 2013 [12-14,27,51-53]. The latest study demonstrated that an 8 years old febrile child was infected by *B. venatorum* from Xinjiang Uygur Autonomous Region and the sequencing results showed that *B. venatorum* is closely related to *B. divergens* [54]. In summary, assuming the cases reported by Hung *et al.* [48] were human babesiosis, about a third of the 27 human babesiosis cases that have been reported in China were occured before 1980 and two thirds thereafter (Figure 3). All of these cases presented with fever and chills and three with severe hepatosplenomegaly. *B. microti* infection may be asymptomatic in about half of children and a quarter of adults [55]. Furthermore, clinical manifestations are typically non-specific, so the diagnosis may have been missed for a long period of time. Recently, a small-scale epidemic of babesiosis was reported in the malaria-endemic areas of the China-Myanmar border in Yunnan province, where the risk of acquiring *Babesia* spp. either from ticks or from human blood products was previously unknown [16].

Most of the cases reported from China were previously diagnosed solely on the basis of detection of parasites in blood smears by microscopy, a method that does not allow for confirmation of species identity. Case reports which included methods for molecular identification of the pathogen, suggest the presence of *Babesia* spp*.* not previously reported from China. Of all reported sporadic human babesiosis cases in China from 1984 to 2014, 16 were identified as *Babesia* species including 14 identified [14,16,27] as *B. microti* and 2 as *B. divergens-*like [54,56]. Meanwhile, based on the information of reported babesiosis cases more than half of these cases were originally infected in south or southeast of China including Taiwan island (Table 2 and Figures 2 and 3).

**Table 2:** **Reported human babesiosis cases in China**

**Province**	**Species**	**Number of reported cases**	**Clinical manifestation**	**Other underlying diseases in patients**	**Reported investigation of reservoir hosts or ticks**	**References**
Chongqing	Unidentified	9	Chills & Febrile	Unclear	No	Hung SL [48]
Yunnan	Unidentified	1	Chills & Febrile	Unclear	No	Li JF *et al.* [51]
	Unidentified	2	Chills & Febrile,	Severe diarrhea	No	Wang HX [13]
		8	arthralgias			
	*B. microti*		Chills & Febrile, arthralgias	History of renal insufficiency caused by malaria	No	Zhou X *et al*. 2013 [16,58]
Inner Mongolia	Unidentified	1	Chills & Febrile, arthralgias	Unclear	Yes	Shi ZB *et al.* [52]
Taiwan	*B. microti*	1	Chills & Febrile, hepatosplenomegaly	Gallstones	Yes	Shih CM *et al.* [27]
Zhejiang	Unidentified	1	Chills & Febrile	Renal failure & renal transplantation	Yes	Su GG *et al.* [12]
	*B. microti*	1	Anemia, Chills & Febrile, hepatosplenomegaly	History of lumpectomy & hysterectomy	Yes	Yao LN *et al.* [14]
Shandong	*B. divergens*	1	unclear	Anemia	No	Qi CH *et al.* [56]
Shanxi	Unclear	1	Chills & Febrile, hepatosplenomegaly	Disc herniation	No	Shanxi Daily news 2013 [53]
Xinjiang	*B. venatorum* (*B. divergens-*like)	1	Anemia & Febrile	No	Yes	Sun Y *et al.* [54]

The presence of *B. microti* and *B. divergens* in ticks and reservoir hosts has been documented (Table 1), but there are no well documented serological studies to confirm the exposure to such agents in tick-infested individuals in China (Table 2). A recent genetic analysis of *B. microti* isolates sampled throughout the USA and Eurasia revealed that this organism represents a genetically diverse species complex [57]. On that basis it has been argued that local variability in the prevalence of *Babesia* spp. in ticks, along with differences in transmissibility and virulence of strains in some geographic areas may explain the lack of clinical cases in humans, despite the local presence of these agents in competent enzootic cycles [47,57]. In addition, lack of medical awareness, may also lead to significant underreporting of human cases in many regions of P.R. China. Correspondingly, the recent occurrence of two ignored indigenous cases of human babesiosis on the China-Myanmar border areas in Tengchong, Yunnan province, is of great medical interest as this is an area where such infections have never been reported. Of these two cases, one had even received transfusion and blood products for treatment of complications. The patient recalled multiple tick bites in the recent past which made this case more complicated [58]. Another confirmed and well documented autochthonous case of *B. microti* infection was reported in 1996 from Taiwan [27]. The patient was a 51-year-old woman from a rural area (Min-Shung, Chia-i Hsien) in southwestern Taiwan and diagnosis of *B. microti* infection was also established by specific PCR and sequencing.

To observe genetic sequence differences of *B. microti* detected in China and other countries, we aligned sequences of 18S rRNA (about 1628-1634 bp) and beta-tubulin gene (about 579 bp) fragments of *B. microti* from a variety of vertebrate hosts (humans, wood mouse, raccoons and rhesus monkey etc.) or from ticks collected in China. These were compared with cases reported in several other countries, such as USA, Spain, Switzerland, Russia, Japan, Korea, and Australia. Phylogenetic trees of maximum-likelihood analysis depicting the relationships of the 18S rRNA gene and beta-tubulin gene of *B. microti* were constructed by MEGA 5.0. Distances were estimated by the Kimura 2-parameter model and the numbers above the branch demonstrate bootstrap support from 1000 replications. The 18S rRNA gene and beta-tubulin gene sequence of *P. falciparum* fragile (JQ627152 and M31205) were included in the trees as outgroups. According to the pathogenesis and the host, *B. microti* has three different clades. It was regarded as a genetically diverse species complex [59]. Clade 1 of *B. microti* contained mostly rodent parasites and also the majority of these strains thought to be zoonotic; Clade 2 contained carnivore parasites and Clade 3 contained rodent parasites which are probably not zoonotic. To identify the isolates reported in China, we carried out the analyses of phylogenetic relationships of *B. microti* strains using maximum-likelihood analyses based on the 18S rRNA partial gene about 1628 bp-1634 bp and beta-tubulin partial gene about 579 bp of *Babesia*.

**Figure 4:**
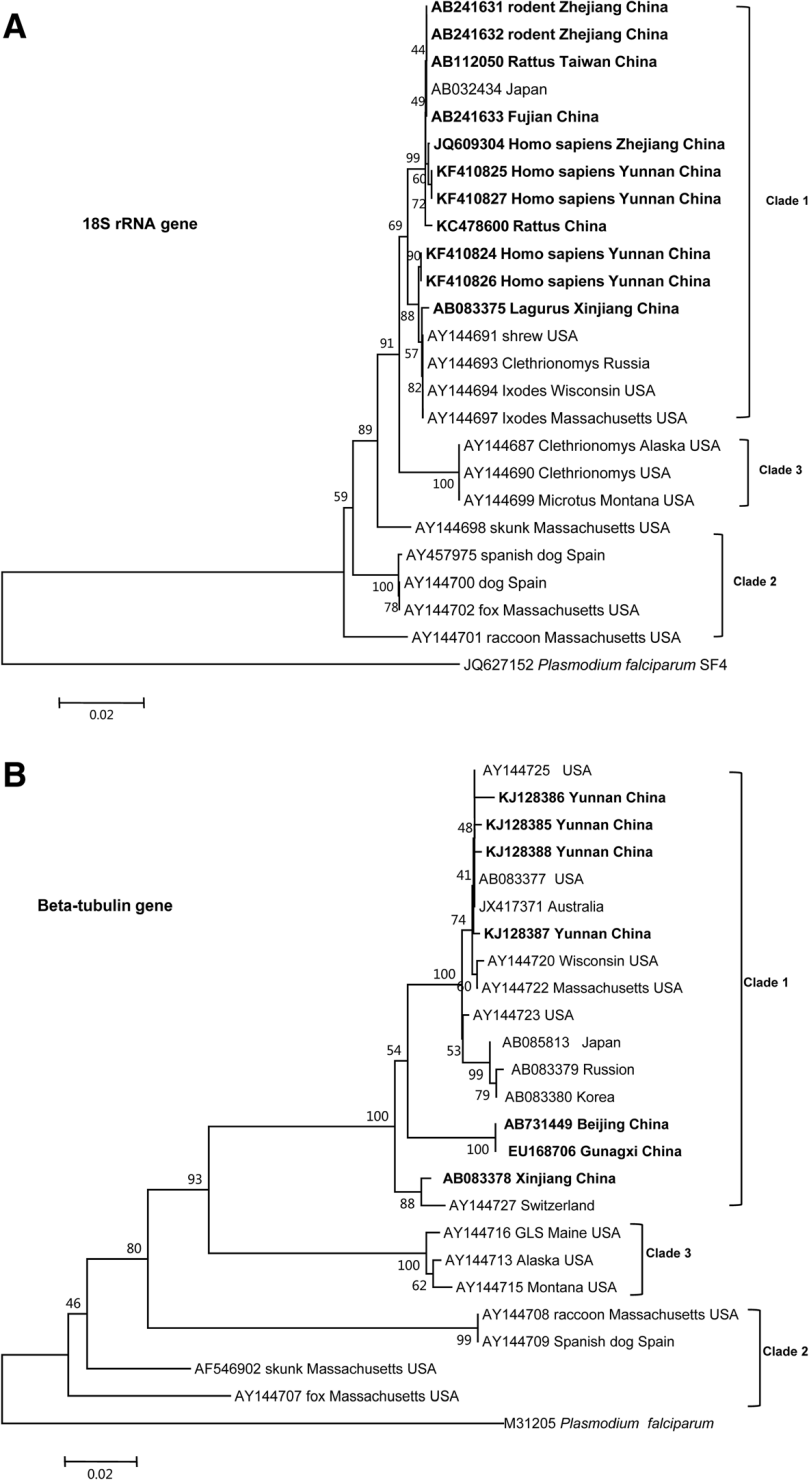
**Phylogenetic relationships of** ***B. microti*** **strains using maximum-likelihood analysis by MEGA 5.0.** Distances were estimated by the Kimura 2-parameter model and the numbers above the branch demonstrate bootstrap support from 1000 replications. The 18S rRNA gene and beta-tubulin gene sequence of *P. falciparum* fragile (JQ627152 and M31205) were included in the trees as outgroups. **A**. based on sequences coding 18S rRNA gene of *B. microti*; **B**. based on sequences coding beta-tubulin gene of *B. microti,* (originated from Goethert HK and Telford SR, 3rd [59], and modified from Zhou X et al. [58].

The sequences of beta-tubulin gene of *B. microti* isolated from raccoon or monkeys from Xinjiang Uygur Autonomous Region, China (AB083378) [43], Guangxi Zhuang Autonomous Region, China (EU168706) [19] and Beijing, China (AB731449) have closer phylogenetic relationships than those (KJ128385, KJ128387) isolated from humans. The sequences of 18S rRNA genes of *B. microti* isolated from humans in Yunnan and Zhejiang provinces, China have the closer phylogenetic relationships with those isolated from Japan and Switzerland. Most of *B. microti* in China appear to be in Clade 1 which was thought to include the zoonotic strains (Figure 4). The alignment results on 18s RNA gene between strains of *B. microti* detected from febrile patients on China-Myanmar border areas, Zhejiang, Taiwan in China, Japan and some strains detected in wildlife rodents were demonstrated in the Additional file 1 and beta-tubulin gene alignment of *B. microti* strains in patients from the China-Myanmar border areas, Australia and *Babesia*-strains detected in neighboring wildlife rodents were listed in the Additional file 2.

## Conclusions

Currently, little data is available on the prevalence of *Babesia* spp. in ticks or rodents in P.R. China [15]. Further sero-epidemiological and molecular epidemiological studies are urgently needed to learn more about the true distribution and medical relevance of these pathogens in various parts of P.R. China.

Clearly, laboratories in babesiosis endemic areas of P.R. China need better access to modern diagnostic methods for a more rapid and reliable microbiological diagnosis in cases of suspected human babesiosis [26,60]. Detection of the asymptomatic but chronically infected blood donors may be useful in preventing -transmitted babesiosis in areas where *Babesia* spp. has zoonotic potential [61,62].

Notably, babesiosis and malaria have similarities and differences in the pathogenesis relating to mechanisms of host cell entry in apicomplexan parasites [23] and the coinfection cases of babesiosis and malaria showed that both diseases appear to be endemic on the China-Myanmar border [42,63,64]. This research approach should pave the way towards successfully controlling both pathogens based on the molecular analysis of erythrocyte invasion.

## Additional files

Additional file 1:
**18s RNA gene alignment results between strains of** ***B. microti*** **detected from febrile patients on China-Myanmar border areas babesiosis Zhejiang in P.R. China, Japan and some strains detected in wildlife rodents.**
Additional file 2:
**Beta-tubulin gene alignment of** ***B. microti*** **strains in patients from the China-Myanmar border areas, Australia and** ***Babesia*** **-strains detected in neighboring wildlife rodents.**
